# Severe Epistaxis Due to Risankizumab: A Case Report

**DOI:** 10.7759/cureus.80627

**Published:** 2025-03-15

**Authors:** Judith P Vinod, Nicholas Gallo, Julio Gonzalez, Christopher Delange, Alejandro Biglione

**Affiliations:** 1 Internal Medicine, Nova Southeastern University Dr. Kiran C. Patel College of Osteopathic Medicine, Fort Lauderdale, USA; 2 Internal Medicine, Wellington Regional Medical Center, Wellington, USA

**Keywords:** il-23 inhibitors, immunotherapy-related adverse events, management of severe epistaxis, risankizumab, s: epistaxis, severe epistaxis

## Abstract

Risankizumab is a novel treatment for plaque psoriasis, approved for treatment in the US in 2019. It is a humanized monoclonal antibody that demonstrates high efficacy and safety, both in short-term and long-term usage. Like many modern medications, risankizumab has some reported adverse effects. The most common of these reported adverse effects, affecting more than 20% of users, is nasopharyngitis. This case report discusses a 76-year-old female patient who presented to the emergency department for severe epistaxis while receiving risankizumab as treatment for plaque psoriasis.

## Introduction

Risankizumab is an interleukin-23 (IL-23) receptor antagonist that is approved in over 70 countries to treat autoimmune conditions [1]. In 2019, the Food and Drug Administration (FDA) approved the drug for treatment of plaque psoriasis in the US [2]. As of 2024, risankizumab is approved for treatment of plaque psoriasis, psoriatic arthritis, and Crohn’s disease, and is currently pending approval for treatment of ulcerative colitis [2]. The pathogenesis of plaque psoriasis, an autoimmune disease characterized by well-demarcated skin lesions covered by silver scales, involves the IL-23/TH17 immune axis [1]. Under normal physiology, the functional IL-23 receptor complex produces intracellular signaling that leads to the production of pro-inflammatory cytokines. Risankizumab binds to IL-23 receptors, blocking downstream signaling and reducing pro-inflammatory cytokines involved in autoimmune diseases [1]. For plaque psoriasis, a 150mg subcutaneous injection is administered on day 0, week four, and every subsequent 12 weeks. Oftentimes, significant clinical improvement is seen as early as after the third injection [2]. However, this benefit is not without fault. Documented adverse effects of risankizumab are nasopharyngitis, upper respiratory tract infections, arthralgia, headache, and hypertension [1-3]. Although these were the more common adverse effects reported, the incidence rate was low and did not worsen with long-term drug exposure [1]. 

Epistaxis, a common affecting 60% of the general population, can be precipitated by certain medications, including immunomodulators like risankizumab [4]. Nosebleeds are more common in children under the age of 10 and adults between 70-79 [4]. The most common etiologies of epistaxis are nose-picking, nasal trauma, environmental changes, viral or allergic rhinitis, and intranasal drug use [4]. Other secondary causes include chronic anticoagulation use, hypertension, and bleeding disorders. These etiologies of epistaxis all lead to weakening and/or damage to the already tenuous blood vessels in the microvasculature which predisposes the vessels to rupture. Most episodes of epistaxis are self-limited and/or controlled by conservative management such as mechanical pressure or ice packs. Epistaxis is classified as anterior or posterior and the location can serve as an indicator of bleed severity. Anterior bleeds are usually caused by the rupture of the veins in the Kiesselbach’s plexus [4]. They tend to be milder and self-limited allowing them to be managed in the outpatient setting. Posterior bleeds are usually caused by the rupture of the posterolateral branches of the sphenopalatine artery [4]. They are typically more severe and can potentially require hospitalization. This article highlights a rare but potentially serious complication of risankizumab therapy, providing insights into its clinical management.

## Case presentation

A 76-year-old female patient with no history of epistaxis presented to the emergency department with intermittent nasal congestion and profuse epistaxis but denied associated symptoms of dizziness, headache, fatigue, syncope, or fever. The patient started having episodes of nasal congestion a month prior to admission followed soon after by intermittent episodes of epistaxis. She had no personal history of bleeding disorders. She also had no history of epistaxis prior to the initiation of risankizumab therapy which was started roughly 10 weeks prior for her psoriasis. Her other past medical history included hypertension, congestive heart failure, chronic obstructive pulmonary disease, and obstructive sleep apnea (OSA) on continuous pressure airway (CPAP) therapy, all of which may influence bleeding risk. Her past surgical history was significant for aortic valve replacement, pacemaker placement, hysterectomy, and bilateral lumpectomy. Her social history was significant for heavy smoking up to one pack of cigarettes per day in the remote past. She admitted to occasionally drinking alcohol and denied any recreational drug use. Family history was relevant for paternal congestive heart failure and maternal stroke. Both her brother and sister had a history of melanoma. There, however, was no family history of bleeding disorders. Her medications included furosemide 40 mg orally daily, losartan 2.5 mg orally daily, metoprolol 100 mg orally daily, pravastatin 20 mg orally daily, aspirin 81 mg orally daily, albuterol 90 mcg/inhalation (two puffs every six hours), umeclidinium-vilanterol 62.5 mcg-25 mcg/inhalation (one puff daily), metformin 500 mg orally daily, duloxetine 20 mg orally daily, gabapentin 300 mg orally daily, letrozole 2.5 mg orally daily, and mirtazapine 7.5 mg orally daily. Among her many therapeutics, aspirin and CPAP therapy could contribute to her bleeding risk. Prior to admission, she had received two doses of risankizumab, with the second one occurring six weeks earlier. Typically patients experience the therapeutic effects after three shots, but our patient reported she had already started to see great improvement.

On the physical exam, vital signs were within normal range. Her chest was clear to auscultation. Her heart exam showed regular rate and rhythm and no murmurs. Her abdomen was soft, nontender, no masses, no organomegaly, bowel sounds normal. Her skin had no active psoriatic lesions. Her ear nose throat exam was significant for profuse epistaxis emanating from her nostrils more prominent from the right.

Her laboratory results are shown in Table 1.

**Table 1:** Laboratory Results of Patient on Risankizumab Therapy Ruling Coagulation Abnormalities or Other Potential Causes of Epistaxis

Lab Value	Patient Value	Normal Range
White Blood Cells (WBC)	13.21	4.50-10.50 x10e3/mcL
Red Blood Cells (RBC)	3.94	4.20-5.50 x10e6/mcL
Hemoglobin	12.8	12.0-16.0 gm/dL
Prothrombin time (PT)	11.5	9.7-11.7 seconds
Activated partial thromboplastin time (aPTT)	24.8	24-35 seconds
International Normalized Ratio (INR)	1.07	0.90-1.20 seconds
Blood Urea Nitrogen (BUN)	33	7-18 mg/dL
Creatinine	0.76	0.55-1.02 mg/dL
BUN/creatinine ratio	43	
Aspartate Aminotransferase (AST)	23	15-37
Alanine Aminotransferase (ALT)	18	12-78

A bilateral nasal tamponade packing was placed in her nares. Initially, the packing controlled her bleeding but the bleeding restarted soon afterwards requiring Neo-synephrine spray and inflation of the device to a higher pressure. The next morning, her hemoglobin dropped down to 10.3. Her epistaxis was completely resolved by the fourth day and she was instructed to follow up with her otolaryngologist and primary care physician within the next two weeks. She was also instructed to return to the emergency department (ED) if profuse bleeding restarted. She was advised to discontinue risankizumab treatments and discuss alternatives with her dermatologist.

## Discussion

Risankizumab is a new innovative immunoglobulin therapy for patients with moderate-severe plaque psoriasis, and other autoimmune diseases. The drug has great efficacy and high safety profile in both short-term and long-term studies [1]. The most commonly reported side effects include pharyngitis, nasal congestion, upper respiratory tract infections (URI), arthralgias, and headaches [1-3]. Most of the reported side effects are mild. According to the physician desk reference, risankizumab has a ~10% incidence rate for both pharyngitis and nasal congestion [5]. The incidence rate of URI is 22.1-36.6%, arthralgias is 0-9.2%, and headaches is 3.5-6.6% [5]. Although the efficacy studies were highly powered; run on large, diverse populations, these trials were done in controlled settings and excluded high-risk patients. Life-threatening epistaxis requiring blood transfusion has not been described. The most common nasal side effects of risankizumab include congestion and rhinitis, which may predispose patients to epistaxis. These conditions, by themselves, could cause epistaxis. Aggravating factors, such as antiplatelet use, CPAP-related dryness, and OSA, should be managed with preventive strategies, including nasal humidifiers and careful evaluation of antiplatelet therapy necessity. Antiplatelet or anticoagulant therapy should be suspended if possible, but unfortunately these agents cannot be suspended most of the time as the diseases they manage are critical. In the case of CPAP therapy, prevention of nose dryness can be achieved via humidifier. 

Patient's normal coagulation profile excludes underlying bleeding diathesis, narrowing the likely cause to medication-induced factors or local nasal pathology. Preliminary clinical studies have observed the interaction between risankizumab and other commonly prescribed drugs such as warfarin, and metoprolol [1]. These studies have concluded that risankizumab is not metabolized by CYP enzyme substrates, nor is it eliminated by the kidneys; therefore, it has minimal drug-drug interactions [1]. The current data suggests that our patient's epistaxis episode is unlikely to have an adverse effect due to a drug-drug interaction. Since risankizmab is a new drug, there is limited data on the pharmacokinetics of the drug in patients with comorbidities like heart failure, chronic kidney failure, or chronic obstructive pulmonary disease (COPD). Therefore, it is difficult to determine whether our patient's comorbidities altered the bioavailability of the drug and how that may have provoked the epistaxis episode. The onset of nasal congestion and epistaxis shortly after initiating risankizumab, followed by resolution after discontinuation, strongly supports a causal relationship.

## Conclusions

Risankizumab is a new IL-23 inhibitor that has been on the market for about five years. Although significant measures have been taken to monitor adverse effects, information remains limited. There is a need for post-marketing surveillance and long-term studies to better understand adverse effects in patients with complex comorbidities who are on polypharmacy. Our case study presents the case of a patient on aspirin for primary prevention with OSA on CPAP therapy who developed severe life-threatening epistaxis requiring hospitalization that occurred soon after starting risankizumab for plaque psoriasis. This case underscores the importance of recognizing severe epistaxis as a potential adverse effect of risankizumab, highlighting the need for careful patient selection and proactive management of risk factors.

## References

[1] Kristensen LE, Keiserman M, Papp K (2023). Efficacy and safety of risankizumab for active psoriatic arthritis: 52-week results from the KEEPsAKE 1 study. Rheumatology (Oxford).

[2] Gordon KB, Lebwohl M, Papp KA (2022). Long-term safety of risankizumab from 17 clinical trials in patients with moderate-to-severe plaque psoriasis. Br J Dermatol.

[3] Calapai F, Ammendolia I, Cardia L, Currò M, Calapai G, Esposito E, Mannucci C (2023). Pharmacovigilance of risankizumab in the treatment of psoriasis and arthritic psoriasis: real-world data from EudraVigilance database. Pharmaceutics.

[4] Tunkel DE, Anne S, Payne SC (2020). Clinical practice guideline: nosebleed (epistaxis). Otolaryngol Head Neck Surg.

[5] (2024). Skyrizi. https://www.pdr.net/drug-summary/?drugLabelId=24336.

